# Bioaccumulation of Trace Elements in Fish From Lake Kivu and Its Potential Risk to Consumers in Rwanda

**DOI:** 10.1002/fsn3.70929

**Published:** 2025-09-15

**Authors:** Valens Habimana, Svetlana Gaidashova, Egide Kalisa, Antoine Nsabimana, Christopher A. Scholz, Charles T. Driscoll

**Affiliations:** ^1^ Department of Chemistry, School of Science, College of Science and Technology University of Rwanda Kigali Rwanda; ^2^ Rwanda Agriculture and Animal Resources Development Board Kigali Rwanda; ^3^ Department of Epidemiology and Biostatistics, Schulich School of Medicine & Dentistry Western University London Ontario Canada; ^4^ Department of Biology, School of Science, College of Science and Technology University of Rwanda Kigali Rwanda; ^5^ University of Rwanda Center of Excellence in Biodiversity and Natural Resource Management, College of Science and Technology Kigali Rwanda; ^6^ Department of Earth and Environmental Science Syracuse University Syracuse New York USA; ^7^ Department of Civil and Environmental Engineering Syracuse University Syracuse New York USA

**Keywords:** bioconcentration factor, estimated daily intake, health risk, pollution index, target hazard quotient

## Abstract

Lake Kivu, located between Rwanda and the Democratic Republic of the Congo, is subject to trace element contamination primarily due to the geological composition of its bedrock, watershed soils, and anthropogenic activities. In this study, we investigated the accumulation of trace elements in 13 fish species from Lake Kivu, with samples obtained from fishermen in Rubavu, Karongi, and Rusizi. The fish samples were digested and analyzed for selected trace element concentrations using atomic absorption spectrophotometry (AAS). The concentrations of trace elements in the fish ranged from 0.07 to 2.53 mg/kg for mercury (Hg), 0.69 to 1.16 mg/kg for cadmium (Cd), 0.28 to 0.76 mg/kg for copper (Cu), 2.58 to 3.83 mg/kg for chromium (Cr), and 0.22 to 0.68 mg/kg for manganese (Mn). The highest bio‐concentration factor (BCF) was observed for mercury. *Oreochromis niloticus* exhibited significantly higher BCFs for mercury compared to other species, with a value of 16,867 L/kg. *Haplochromis scheffersi* also displayed a high BCF for mercury at 3533 L/kg, followed by *Labeo victorianus* with a BCF of 2867 L/kg. Mercury (Hg) posed a potential risk for adults in 77% of the fish species analyzed, as indicated by target hazard quotient (THQ) values exceeding 1. Additionally, Cd, Cr, and Hg posed potential risks for children in over 75% of the fish species analyzed. Continuous monitoring of trace element sources and concentrations in the water column and fish of Lake Kivu is urgently needed to assess contamination sources and exposure levels.

## Graphical abstract

Hg, Cd and Cr were detected in 13 fish species with Hg showing the highest BCF in *Oreochromis niloticus*. Hg used a risk to adults 77% of fish species analyzed while Cd, Cr and Hg posed risk to children in over 75. There is an urgent need to mitigate potential health hazards to consumers.

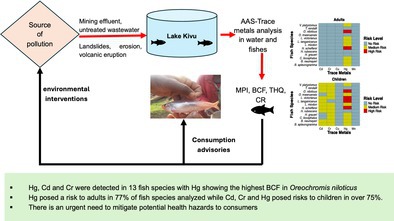

## 1 Introduction

The current agricultural policy in Rwanda aims to increase fish production, with a particular focus on aquaculture and cage farming in lakes (RAB 2020). Lake Kivu, the deepest and largest lake in Rwanda, has the highest potential for increasing fish production (Hoang Le et al. 2016). Lake Kivu is a stratified lake with a tectonic origin; its properties are affected by the magmatic activities, which have implications on short‐term processes such as inflow, outflow, and lake levels (Wood et al. 2015). Unlike other deep Great Lakes of Africa, where stratification is driven by warmer, less dense waters sitting atop cooler, deeper waters, stratification in Lake Kivu is primarily driven by the higher solute concentrations of the hypolimnion, resulting from several sub‐aquatic springs entering the lake (Schmid and Alfred 2012). These unique geological conditions also affect its fish diversity and species richness (Snoeks et al. 2012). Compared to other lakes in the eastern region, Lake Kivu has poor fish diversity, with fewer than 30 fish species (Villanueva et al. 2008).

Fishing is a crucial activity for the communities residing along the shores of Lake Kivu, providing a significant and regular source of income and protein through fish consumption, with *Limnothrissa miodon* being the major commercial fish in the lake (Balagizi et al. 2018). Studies have not been conducted to provide details on the frequency and quantity of fish consumed by the populations in Rwanda (de Bruyn et al. 2021). However, a survey conducted by the Rwanda National Institute of Statistics indicated the highest rates of fish consumption (16%–24% of households) in Rwanda occurred for communities adjacent to Lake Kivu, with the highest fish consumption among the poorest wealth‐class households (31%) compared to other wealth classes (1%–7%) (NISR 2018).

Exposure to trace elements through contaminated food remains a significant public health concern, as environmental pollution can lead to toxic elements accumulation even in widely consumed food products (Abbas et al. 2024; Erdoğan et al. 2024). Trace elements contamination in food chains, including fish, is an important public health issue where bioaccumulation from polluted aquatic environments can result in dietary exposure (Abbas 2024; El‐Shorbagy et al. 2025). Despite the reliance of the local community on Lake Kivu fish as a primary protein source, studies have not been conducted on trace element contamination in Lake Kivu fish. A study was conducted in 2011 to examine trace elements in the water of Lake Kivu, finding concentrations exceeded the permissible limits (Olapade 2011). Building on this initial investigation, we conducted a more detailed study of trace elements in the waters of Lake Kivu, which revealed that Cd, Pb, Cu, Cr, Mn, and Hg were above the maximum allowable concentrations for class III surface waters intended for fish consumption and recreational use (Nsabimana et al. 2020).

The present study extends this work by investigating trace element bioaccumulation in fish species from the lake. When the rate of uptake of trace elements by organisms exceeds the rate of elimination, concentrations in organisms can accumulate to values much higher than water (Mendis and Najim 2015). Aquatic organisms at higher trophic levels are likely to accumulate trace elements to a greater degree than those at lower trophic levels, leading to trophic enrichment and exposure in humans who regularly consume fish (Kalkan et al. 2015).

The consumption of fish contaminated with trace elements poses several health risks to humans, including neurological, cardiovascular, renal, reproductive, and carcinogenic effects (Hossain et al. 2024). The specific risks depend on the type and concentration of the trace elements, the duration of exposure, and the health condition of the individual exposed (Jaishankar et al. 2014).

Hg can cause particularly severe neurological damage, particularly in developing fetuses and young children (Dack et al. 2022). All forms of Hg are toxic and each has its unique toxicological effects (Yang et al. 2020).

Pb exposure is known to cause cognitive deficits and behavioral problems, especially in children, and chronic exposure can lead to kidney damage and impaired renal function (Ruebner et al. 2019).

As is a naturally occurring metalloid essential in trace amounts for human life but becomes toxic at higher levels (Yüksel et al. 2010). As is associated with various cancers and cardiovascular diseases (Rahimzadeh et al. 2017).

Cd is not essential for humans and is toxic at low concentrations. The health effects of consuming high levels of Cd include reproductive failure, damage to the central nervous system, psychological disorders (Zuluaga et al. 2015) and immune system and kidney disorders (Ishak et al. 2020).

Cr is an essential element to humans involved in the functioning of insulin and has an important role in the metabolism of lipids. However, not all the Cr forms are safe; only Cr(III) is essential for human metabolism and only needed in trace amounts (Saha et al. 2016). Cr(VI) is highly toxic even at low concentrations. Exposure to high levels of Cr can lead to gastrointestinal distress, liver damage, respiratory issues, and increased cancer risk (Gaetke et al. 2014).

Cu is an essential element for mammals, as a component of metalloenzymes and playing the role of electron donor or acceptor processes (Taylor et al. 2007). Cu is only required in trace amounts, and at high concentrations may produce free radicals, which damage the DNA and neurons (Desai and Kaler 2008). Cu in excess also displaces other metal cofactors in metalloenzymes; therefore, disturbing the normal function of the body (Stern 2010). Mn exposure can result in neurotoxicity, presenting symptoms similar to Parkinson's disease (Lu et al. 2023).

In this study, we aim to fill a critical gap in knowledge regarding the bioaccumulation of trace elements in fish from Lake Kivu and its implications for public health in Rwanda. The study seeks to quantify the concentrations of selected trace elements in different fish species from the lake, identify possible sources of trace elements contamination, and evaluate the potential human health risks associated with the consumption of contaminated fish. Furthermore, we aim to provide recommendations for safe fish consumption in order to safeguard public health and ensure the long‐term sustainability of Lake Kivu's fisheries.

## 2 Materials and Methods

### 2.1 Study Area Description

**FIGURE 1:**
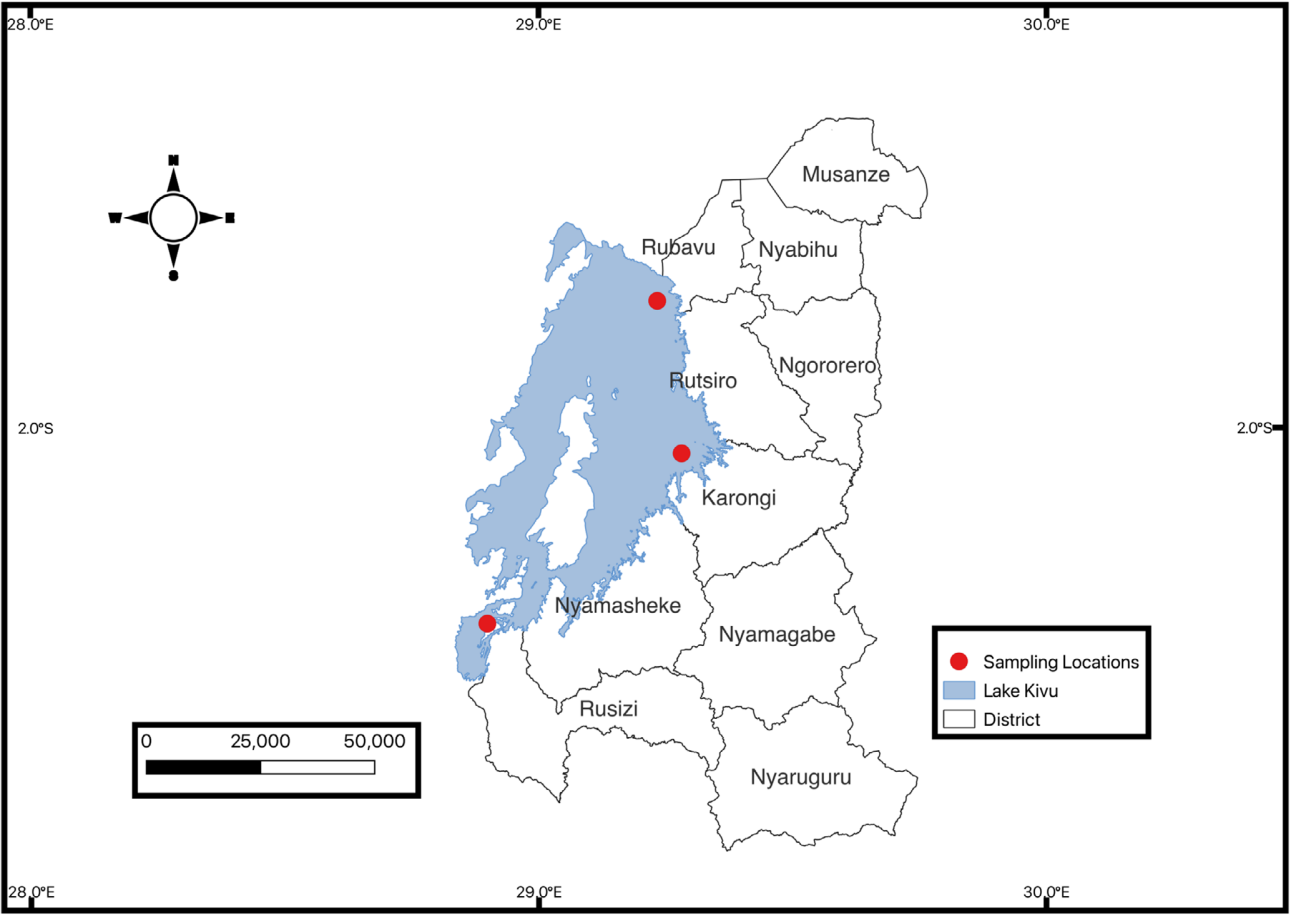
Map of Lake Kivu with fish sampling locations. This map of Lake Kivu highlights the fish sampling locations, marked by red dots. Sampling was conducted in three key districts: Rusizi, Rubavu, and Karongi, which are known for fishing activities and contribute to the fish supply in Rwanda.

Fish samples were collected from Lake Kivu (Figure 1). The lake has a large surface area (2386 km^2^) and depth (485 m), and it is a permanently stratified tropical rift lake of volcanic origin (Bärenbold et al. 2022). The region has an altitude range of 1470 to 2200 m above mean sea level and experiences average temperatures ranging from 16°C to 21°C, with annual rainfall between 1100 and 1500 mm (Murera and Donald 2021). Fishermen on Lake Kivu are organized into fishing cooperatives, which sell their catch to a fishing project that manages the transportation, trade, and processing of the fish. Fish samples were collected from three sampling sites, namely Rusizi (2° 31.91′ S 28° 95.72′ E); Rubavu (1° 41.154′ S 29° 8.292′ E) and Karongi (1° 94.12′ S 29° 12.28′ E) (Figure 1). The sampling sites were chosen in proximity to areas with significant anthropogenic activities, including urban centers, as well as locations receiving tributaries from rivers that drain mining areas. These sites were selected to represent varying levels of contamination due to different sources of anthropogenic influence around the lake. Furthermore, these same sites were used in our previous studies of trace element pollution in Lake Kivu's water, ensuring consistency and comparability between the studies (Nsabimana et al. 2020).

The Rubavu site was located near Rubavu Secondary City in Rwanda, the most urbanized among the three cities, with a high population density of 1614 inhabitants/km^2^ (NISR 2023). This site is in proximity to significant industrial activities, including breweries, factories, and commercial trade, due to its border location with Goma (Democratic Republic of Congo) (Fabrice Amisi et al. 2023). Erosion and landslides occur frequently, and urban runoff is likely more pronounced in this area due to the prevalence of paved surfaces and extensive infrastructure (REMA 2023). While some mining activities occur in the surrounding areas, they are less dominant compared to Rusizi (Dusengemungu et al. 2023). The Rusizi site was situated near Rusizi Secondary City, which is moderately urbanized compared to Rubavu but serves as an important commercial area due to its border with Bukavu (Democratic Republic of Congo) (Nene Morisho Mwanabiningo 2015). Mining activities occur in the surrounding watershed, and urban runoff is evident but less pronounced than in Rubavu (ABAKIR 2020). The Karongi site was located near a less urbanized city compared to Rubavu and Rusizi; urban runoff at this site was minimal due to the lower level of urbanization (NISR 2023).

### 2.2 Sample Collection

Due to the methane production and accumulation in lower waters, Lake Kivu has a relatively poor fish fauna compared to other African Great Lakes, with only 28 fish species reported in the lake and its tributaries (Kisekelwa et al. 2023). The low fish diversity is largely attributed to the geological history of the lake, including catastrophic events such as the mid‐Miocene uplift of the East African region, which is responsible for the formation of the lake at high altitude (Nsabimana et al. 2021). The 13 fish species examined in this study were collected in July 2022 from the commonly captured fish species by permitted fishermen; therefore, no additional permit was required for this study. The sampling process adhered to best practices recommended by the Rwandan government for sustainable fish harvesting. The collected samples were kept in sealed plastic containers with ice and stored in a freezer at −12°C prior to chemical analysis. The samples were measured for their length (cm) using a Vernier caliper, and their weights were recorded (g) using an electronic balance. The morphometric characteristics of the studied fishes, including their common and scientific names, family, length, weight, feeding habits, and habitat type are documented in a previously published study on the morphometric characteristics of important fish species from the Rwandan side of Lake Kivu (Nsabimana et al. 2020).

### 2.3 Reagents

All chemicals utilized in the analytical procedures were of ultrapure grade. Nitric acid (68%) and perchloric acid (70%) were obtained from Fisher Scientific (UK). Element standard solutions were obtained from Sigma‐Aldrich (UK) while the certified reference materials were sourced from the National Research Council Canada (NRC).

### 2.4 Sample Preparation

The fish samples were washed with distilled water and dried for 24 h to constant weight, in an oven at 105°C. After drying the fish sample in the oven, the bones and scales of the dried fish samples were removed, leaving only the muscle, head, tail, eyes, and gills of the fish sample. The remaining parts of the fish sample were milled with a mortar and pestle. For each sample, 2 g of homogenized fish tissue was mixed with 5 mL of HNO_3_ and 2 mL of HClO_4_ and digested using a block digester for 30 min at 85°C. After completing the digestion, the residue was allowed to cool and filtered into a 50 mL volumetric flask using a 0.45 μm micropore membrane filter. The solutions were then made up with distilled water to 50 mL and analyzed for trace element concentration.

### 2.5 Sample Analysis Procedure

The samples were analyzed by atomic absorption spectrophotometry (AAS), Shimadzu AA‐6800, Japan. AAS is a reliable method for the accurate determination of Pb, As, and other trace elements due to its sensitivity and precision (Yüksel et al. 2010; Yüksel et al. 2016). In Table 1 the wavelength of detection, calibration range, correlation coefficients (*R* ^2^), detection limits (LOD) and quantification limits (LOQs) for each of the trace elements analyzed are summarized. The recovery experiments, relative standard deviations, and calibration curves were evaluated to ensure the reliability of the method, following the approach outlined by Yüksel et al. (2016). To verify the analytical method, a repeatability test was performed by spiking the samples with various concentrations of trace elements (Table 2). The accuracy of the analytical procedure was checked by using certified reference materials for trace elements obtained from the National Research Council Canada (NRC).

**TABLE 1:** Calibration range, correlation coefficients, limits of detection (LOD), and limits of quantification (LOQ) values.

Metals	Wavelength (nm)	Calibration range (mg/L)	*R* ^2^	LOD	LOQ
Cu	324.7	0.02–5	0.9987	0.01	0.02
Cr	357.9	0.01–3	0.9984	0.002	0.01
Cd	228.8	0.01–2	0.9991	0.005	0.01
Hg	253.7	0.01–1	0.9989	0.005	0.01
Mn	279.5	0.005–2	0.9993	0.002	0.005
Pb	217	0.01–5	0.9995	0.005	0.01
As	193.7	0.01–2	0.9987	0.005	0.01

*Note:* The calibration range, correlation coefficients (*R* ^2^), limits of detection (LOD), and limits of quantification (LOQ) for trace elements analyzed in this study. The calibration range defines the concentration limits within which the instrument provides a linear response for each element. The correlation coefficient (*R* ^2^) values, indicate linearity of the calibration curves. The LOD represents the lowest concentration of element that can be reliably detected, while the LOQ signifies the minimum concentration that can be quantified with acceptable precision and accuracy. The wavelengths (in nm) used for each element detection are also provided.

Abbreviations: LOD, Limits of detection; LOQ, Limits of quantification; *R* ^2^, Correlation coefficient.

**TABLE 2:** Recovery of trace elements from fish muscle.

Metals	Spiked values (mg/kg)	Measured value (mg/kg)	% recovery
Cu	4	3.305	82.6
Cr	5	4.315	86.3
Cd	2	1.705	85.3
Hg	1	0.812	81.2
Mn	2	1.746	87.3
Pb	3	2.805	93.5
As	1	0.964	96.4

*Note:* The recovery efficiency of trace elements from fish muscle samples. Recovery was determined by spiking known concentrations of trace elements into the samples and comparing the measured concentrations to the expected values.

#### 2.5.1 Calculation of Pollution Index (PI)

The Pollution Index was calculated using the formula below:

$$\mathrm{PI}={\left(M_{1}\cdot M_{2}\cdot M_{3}\cdot M_{4}\cdot M_{5}\right)}^{1/5}$$

*M* is the concentration of trace elements in mg/kg. The PI for all fish species was compared to evaluate the fish species with a high Pollution Index (Girgis et al. 2019).

#### 2.5.2 Calculation of Bio‐Concentration Factor (BCF)

The BCF was evaluated as the net results of the uptake, distribution, and elimination processes of trace elements from lake water. The bio‐concentration factor was calculated as the ratio between the measured concentrations in fish to the measured average concentration in lake water (Ahmed et al. 2019). The concentrations of trace elements in lake water used for this study were previously reported, based on samples collected from the surface to a depth of 40 m. From this earlier study, the average concentrations of Cd, Cu, Cr, Mn, and Hg in the water samples were 0.0084, 0.00642, 0.177, 0.669, and 0.00015 mg/L, respectively (Nsabimana et al. 2020).

$$\mathrm{BCF}=\frac{\;\text{Average concentration of trace elements in the fish in}\;\mathrm{mg}/\mathrm{kg}}{\text{Average concentration of trace elements in the water in}\;\mathrm{mg}/L}$$

### 2.6 Health Risk Assessment

The potential health implications of consuming the examined fish species were estimated using the following metrics: Estimated Daily Intake (EDI), Target Hazard Quotients (THQ), and Target Cancer Risk (TR) of the trace elements investigated. Health risks for adults and children were evaluated separately, based on the hypothesis that children are more susceptible to the effects of contaminants than adults (Kaba et al. 2023).

### 2.7 Calculation of the Estimated Daily Intake

The estimated daily intake of fish per person per day was calculated using the survey data on Rwanda nutrition analysis and market and gender analysis, an integrated approach towards alleviating malnutrition among vulnerable populations in Rwanda (RAB 2015). The data were published by the Ministry of Agriculture in partnership with the Ministry of Health in Rwanda in 2015 through the Rwanda Agriculture Board. The consumption rate of fish was estimated to be 40 g/day for adults and 5.6 g/day for children. The concentration of trace elements, to which people may be exposed via fish consumption, was determined in the laboratory. The EDI was calculated in mg/day‐kg body weight by multiplying the trace element concentrations (in mg/kg) by the fish consumption rate (in kg/day) divided by the body weight (in kg) of the person of interest. A correction factor was included to represent the edible fraction of the fish, accounting for the portion of the fish mass that is actually consumed. In this study, 𝐸 was set to 1 since only edible muscle tissue, after removing bones and other inedible materials, was analyzed in the laboratory.

$$\mathrm{EDI}=\frac{\text{trace element concentration in fish tissue}\left(\frac{\mathrm{mg}}{\mathrm{kg}}\right)\cdot \text{fish consumption rate}\left(\mathrm{kg}/\mathrm{day}\right)}{\text{body weight}\;\left(\mathrm{kg}\right)}\cdot E$$

The body weight (bw) was assumed to be 70 kg for adults and 5 kg for children.

### 2.8 Calculation of Target Hazard Quotient (THQ) and Hazard Index (HI)

To evaluate the risk of trace element exposure from fish consumption in humans, the target hazard quotient (THQ) was used. This approach was also used by several other studies (Gutiérrez et al. 2017; Kumar Kundu et al. 2017; Kaba et al. 2023; Artar et al. 2024; Yin et al. 2024). The THQ represents the ratio of the exposure level of a single substance over a specific period to the reference dose (RfD) for that substance, based on a similar exposure duration (Kaba et al. 2023). The THQ is designed to indicate whether the exposure level is below a threshold where adverse health effects are unlikely; even for vulnerable populations, if the exposure level exceeds this threshold, there is a potential concern for non‐carcinogenic effects (Andemo Kotacho et al. 2024).

THQ was calculated as the ratio of estimated daily intake (EDI) to the reference dose (RfD) according to the formula below:

$$\mathrm{THQ}=\frac{\mathrm{EDI}}{\text{ARfD}}$$

The ARfD is the accepted reference dose for the particular trace element in mg/kg bw. The US‐EPA has established the following oral reference doses for trace elements: 0.001 for Cd, 0.04 for Cu, 0.003 for Cr, 0.14 for Mn, and 0.0001 for Hg mg/kg bw (USEPA 2021).

After the calculation of the target hazard quotient, the hazard index was calculated as the sum of the individual target hazard quotients. The calculation of HI was performed as per Ahmed et al. (2019) using the formula below.

$$\mathrm{HI}=\sum_{n=1}^{i}\mathrm{TH}Q_{n}$$

### 2.9 Calculation of Target Cancer Risk (TCR)

The target cancer risk (TCR) was calculated to evaluate the hazard for the trace elements investigated to cause cancer. Instead of using the accepted reference dose used to evaluate the hazard as non‐cancer effects caused by the trace elements, an oral cancer slope factor (CPSo) was used to calculate the target cancer risk. The oral slope factor indicates the minimum dose of particular trace elements, which may not probably result in cancer when exposed to it during the lifetime (Isa et al. 2015). The target cancer risk was calculated as per the formula below.

$$\mathrm{TCR}=\frac{C\cdot \mathrm{FIR}\cdot \mathrm{EFr}\cdot \mathrm{ED}\cdot \mathrm{CP}S_{O}}{\mathrm{bw}\cdot \mathrm{AT}}$$

where *C* is the concentration of a trace element in mg/kg of fish tissue wet weight, FIR represents the rate of food ingestion in kg/day‐person, estimated to 0.04 kg/day, EFr is the exposure frequency, which is assumed to be 365 days per year. The ED is the duration of the exposure, which is the life expectancy in Rwanda (67.8 years), bw is the body weight (70 kg) for an adult, and AT is the average exposure time, which is 365 days * 67.8 years. The US‐EPA has set only the oral cancer slope factor for the following trace elements: 1.5 mg/kg‐day for arsenic, 0.0085 mg/kg‐day for lead, and 6.3 mg/kg‐day for Cd (USEPA 2021).

### 2.10 Statistical Analysis

Variations in trace element concentrations among fish species were evaluated using one‐way analysis of variance (ANOVA) in R (version 4.3.2), with species as the independent variable and trace element concentration as the dependent variable. Significant differences between species were further examined using Tukey's Honestly Significant Difference (HSD) post hoc test. A significance threshold of *p* < 0.05 was applied for all analyses (Afifi et al. 2024; El‐Kady et al. 2025).

## 3 Results and Discussion

This study presents a detailed evaluation of trace elements bioaccumulation in fish species from Lake Kivu and its associated health risks to consumers in Rwanda. Trace elements concentrations were analyzed across various fish samples, and the extent of trace elements contamination was assessed using the Pollution Index (PI). The Bio‐concentration Factor (BCF) was calculated to assess the fish's capacity to bioaccumulate trace elements, providing insight into the environmental burden. Additionally, human health risk assessments were performed using the Target Hazard Quotient (THQ) to estimate noncarcinogenic risks, while Carcinogenic Risk (CR) estimates were specifically calculated for Cd. Collectively, these findings illustrate the potential exposure to trace elements through fish consumption and their public health implications.

### 3.1 Trace Element Concentrations in Fish Samples From Lake Kivu

In this section we present data on trace element concentrations (mg/kg) and pollution index (PI) for various fish species in Lake Kivu (Table 3). A comparison of obtained results of trace elements in fishes from Lake Kivu with other studies is shown in Table 4, while the maximum permissible limits of trace elements in fishes are summarized in Table 5. The trace elements analyzed include Pb, As, Cd, Cu, Cr, Mn and Hg. Both Pb and As concentrations were below the limit of quantification (LOQ) for all fish species. The data indicated a significant variability in trace element accumulation among different fish species, with some species exhibiting higher trace element concentrations and pollution indices. The variations of trace elements in fish tissue may be explained by different accumulation patterns due to variations in trophic level, different preferences in food sources, and different uptake and elimination mechanisms for specific contaminants (Eldeen and Mansour 2020).

**TABLE 3:** Trace element concentrations (mg/kg) and pollution index (PI) for fish species in Lake Kivu.

Scientific names	Local names	Cd	Cu	Cr	Mn	Hg	As	Pb	MPI
*Labeo victorianus*	Indugu	0.97 ± 0.48	0.41 ± 0.25	2.97 ± 1.65	0.35 ± 0.09	0.43 ± 0.06	< LOQ	< LOQ	0.71
*Tilapia rendalli*	Indoba	1.01 ± 0.07	0.46 ± 0.31	3.28 ± 0.71	0.40 ± 0.07	0.08 ± 0.02	< LOQ	< LOQ	0.55
*Barbus neumayeri*	Ingege	1.08 ± 0.06	0.36 ± 0.26	3.13 ± 1.02	0.30 ± 0.09	0.26 ± 0.03	< LOQ	< LOQ	0.62
*Clarias liocephalus*	Isembe	0.89 ± 0.40	0.28 ± 0.08	3.17 ± 0.65	0.22 ± 0.03	0.20 ± 0.04	< LOQ	< LOQ	0.51
*Oreochromis niloticus*	Tilapia	1.11 ± 0.64	0.76 ± 0.48	3.83 ± 1.1	0.68 ± 0.26	2.53 ± 0.82	< LOQ	< LOQ	1.41
*Limnothrissa miodon*	Isambaza	0.93 ± 0.7	0.33 ± 0.07	2.85 ± 0.84	0.27 ± 0.07	0.20 ± 0.05	< LOQ	< LOQ	0.54
*Varicorhinus platystomus*	Gakara	0.92 ± 0.01	0.32 ± 0.13	2.92 ± 1.01	0.27 ± 0.08	0.19 ± 0.05	< LOQ	< LOQ	0.54
*Barbus apleurogramma*	Imyungu	1.16 ± 1.03	0.36 ± 0.18	3.05 ± 1.28	0.31 ± 0.08	0.23 ± 0.02	< LOQ	< LOQ	0.62
*Oreochromis mweruensis*	Bwiza	0.85 ± 0.34	0.33 ± 0.11	2.73 ± 0.94	0.27 ± 0.03	0.16 ± 0.03	< LOQ	< LOQ	0.51
*Lamprichthys tanganicanus*	Rwandarushya	0.94 ± 0.20	0.35 ± 0.14	2.58 ± 0.31	0.29 ± 0.05	0.36 ± 0.05	< LOQ	< LOQ	0.62
*Haplochromis graueri*	Mupfumu	0.69 ± 0.39	0.33 ± 0.06	2.81 ± 0.83	0.27 ± 0.10	0.20 ± 0.03	< LOQ	< LOQ	0.51
*Haplochromis rubescens*	Gishoga	0.94 ± 0.28	0.38 ± 0.11	2.97 ± 1.09	0.32 ± 0.08	0.07 ± 0.02	< LOQ	< LOQ	0.47
*Haplochromis scheffersi*	Baraka	0.98 ± 0.59	0.35 ± 0.07	2.89 ± 0.80	0.29 ± 0.04	0.53 ± 0.06	< LOQ	< LOQ	0.69

*Note:* The concentrations of trace elements in various fish species collected from Lake Kivu, expressed in mg/kg. The fish species are listed with their scientific names alongside their corresponding local names. The pollution index (PI) is calculated as an aggregate measure of contamination levels across different trace elements for each species, providing an indication of overall trace elements accumulation. Trace element concentrations below the limit of quantification are denoted as < LOQ. The data highlight species‐specific differences in trace element accumulation, which may have implications for human consumption and environmental monitoring.

Abbreviations: LOQ, Limit of Quantification; PI, Pollution Index.

**TABLE 4:** Comparison of trace element concentrations in fish species from Lake Kivu with other studies.

Pb	Cd	Mn	Cr	Hg	Cu	AS	References
< LOQ	0.661–1.431	0.204–1.455	2.422–6.406	0.029–5.413	0.255–1.592	< LOQ	Present study
NM	NM	NM	NM	0.24–0.44	NM	NM	Emmanuel and Samuel (2009)
NM	NM	NM	NM	0.03–0.64	NM	NM	Barone et al. (2021)
0.73–3.24	0.02–0.51	0.26–1.26	NM	NM	3.01–17.47	NM	Zaqoot et al. (2017)
NM	NM	0.22–1.9	NM	NM	0.04–1.2	NM	Gurganari et al. (2024)
0.21–0.88	0.03–0.12	0.10–0.29	NM	NM	0.22–0.36	NM	El‐Moselhy et al. (2014)
4.35–8.03	0.87–1.35	NM	4.71–8.98	0.08–0.41	14.00–31.80	0.17–0.28	Saha et al. (2021)
0.2–0.68	NM	ND‐7.1	BDL −9.69	0.72–2.9	30.29–48.59	BDL	Hossain et al. (2022)
0.025–0.385	NM	NM	0.059–1.605	NM	NM	NM	Mohiuddin et al. (2022)
0.04–0.33	0.004–0.021	NM	0.07–0.28	0.01–0.05	0.21–0.884	0.03–0.2	Zheng et al. (2007)
0.052–0.087	0.023	NM	0.048–0.083	NM	0.036–0.097	NM	Rajeshkumar and Li (2018)
NM	NM	0.2–0.4	NM	NM	0.14–0.24	NM	Rajkowska and Protasowicki (2013)
1.03–7.79	NM	NM	NM	NM	0.25–3.71	NM	Mapenzi et al. (2020)
3.05–5.12	1.60–2.48	19.81–37.18	0.94–2.11	NM	18.71–43.61	3.25–7.31	Mahboob et al. (2014)
0.597–1.124	ND	0.384–0.385	ND‐0.154	NM	0.071–0.407	NM	Andemo Kotacho et al. (2024)
0.074–0.084	0.003–0.004	NM	NM	NM	NM	NM	Ishak et al. (2020)

*Note:* A comparative analysis of trace element concentrations in fish species from Lake Kivu and values reported in previous studies conducted in various aquatic environments. The concentration ranges are given in mg/kg where < LOQ indicates metal concentrations below the limit of quantification, while NM denotes cases where the trace element was not measured, and ND indicates the trace element was not detected, while BDL indicates the trace element below the detection limit.

**TABLE 5:** Maximum concentration limits of trace elements in fish.

Trace metals	Maximum limits	Source	References
As	0.5 mg/kg	Codex Alimentarius	CODEX (2009)
Cd	0.05 mg/kg	European Union Directive	EU‐EC (2005)
Pb	0.2 mg/kg	European Union Directive	EU‐EC (2005)
Hg	0.5 mg/kg	European Union Directive	EU‐EC (2005)
Cu	3 mg/kg	FAO/WHO	FAO/WHO (1989)
Cr	0.15 mg/kg	FAO/WHO	FAO/WHO (1989)
Mn	1 mg/kg	FAO/WHO	FAO/WHO (1989)

*Note:* The maximum allowable concentrations of trace elements in fish as established by international regulatory bodies, including the Codex Alimentarius, the European Union Directive, and FAO/WHO. The values are given in mg/kg, with regulatory references provided for each trace element.

Abbreviation: FAO/WHO, The joint Food and Agriculture Organization and the World Health Organization.

The highest trace element pollution values in Lake Kivu fish were found in Rusizi, indicating significant trace element accumulation in this area. Rubavu exhibited moderately high pollution values, while Karongi had comparatively lower pollution values, suggesting relatively lower supply of trace elements to this region of the lake.

Cd concentrations were elevated in Rubavu and Rusizi, likely due to contributions from industrial discharge and mining runoff. Cd is known for its high toxicity, even at low concentrations, and can bioaccumulate, leading to adverse effects such as significant damage to tissue structure and function, as well as disrupting the antioxidant defense system, reproductive regulation, and immune function in fish and other aquatic species (Liu et al. 2022).

Cu levels were relatively consistent across all sites but were slightly higher in Rusizi, possibly influenced by mining activities. While Cu is an essential trace element for aquatic life, excessive concentrations can disrupt enzyme function and impair the gill function of fish, leading to respiratory distress and increased mortality rates (Malhotra et al. 2020).

Cr concentrations were highest in Rusizi, aligning with known mining activities in the region. Cr, particularly in its hexavalent form (Cr(VI)), is highly toxic and carcinogenic, affecting the survival and development of aquatic organisms and potentially accumulating in sediments, prolonging its environmental impact (Velma et al. 2009).

Similarly, Mn reached its peak levels in Rusizi, further supporting the influence of mining operations. Though Mn is a necessary micronutrient, excessive amounts can cause neurological damage in fish and alter microbial communities in water bodies, disrupting nutrient cycling (Harford et al. 2015; Ali et al. 2021).

Notably, Hg concentrations in Rusizi were exceptionally high, suggesting potential contamination from mining activities or industrial waste discharge. Hg is particularly concerning due to its transformation into methylmercury, a highly toxic compound that bioaccumulates and biomagnifies through the food web. Small aquatic organisms absorb Hg, which is then transferred to larger predators, ultimately posing significant health risks to fish‐consuming wildlife and humans (Driscoll et al. 2013).

The cumulative effects of trace element contamination in these areas threaten aquatic biodiversity, weaken ecosystem resilience, and increase the risk of toxic exposure in local populations relying on these water sources. If left unmanaged, persistent trace element pollution could lead to long‐term ecological imbalances, declining fish populations, and heightened risks of trace element poisoning in humans through dietary intake (Phaenark et al. 2024; Tepe et al. 2024).

### 3.2 Pollution Index (PI) Analysis

The Pollution Index (PI) provides an overall indication of the level of trace element contamination in each fish species (Moussa et al. 2022). A higher PI corresponds to a greater degree of trace element contamination (Ramish et al. 2024). A PI value of less than 1 indicates a safe degree of contamination, values between 1 and 2 indicate slight contamination, 2–3 indicate moderate to severe contamination, 3–5 indicate severe contamination, and values greater than 10 indicate extreme contamination (Abbas et al. 2024).

Among the species analyzed, *Oreochromis niloticus* had the highest PI of 1.41, indicating slight pollution. The lowest PI was observed in *Haplochromis rubescens* , with a value of 0.47, suggesting lower contamination. This pattern may be explained by the omnivorous feeding behavior of *O. niloticus* , which consumes a diverse diet including plankton, detritus, and benthic organisms, potentially increasing its exposure to metal‐contaminated food sources (Ouro‐Sama et al. 2021). Additionally, *O. niloticus* often inhabits shallow waters near river mouths and urban areas, where trace element contamination from anthropogenic sources is more pronounced, such as wastewater discharge and mining runoff (Chavan and Yakupitiyage 2012).

### 3.3 Cadmium (Cd)

The average concentration of Cd across the fish species was 0.96 mg/kg, with the highest concentration observed in *Barbus apleurogramma* (1.16 mg/kg) and the lowest in *Haplochromis graueri* (0.69 mg/kg).

Analysis of variance (ANOVA) revealed no statistically significant differences in cadmium (Cd) concentrations among the 13 fish species examined (*F* = 0.182, *p* = 0.998), indicating that species identity had no detectable effect on Cd levels in this dataset. The combination of a very high *p*‐value and a low *F*‐statistic suggests that the observed variation in Cd concentrations is largely attributable to within‐species variability rather than differences between species. Tukey's HSD post hoc comparisons supported this conclusion, as all pairwise contrasts exhibited small mean differences, wide confidence intervals encompassing zero, and adjusted *p*‐values approaching 1. These findings collectively indicate that Cd accumulation was relatively consistent across species under the environmental and biological conditions of the present study.

CD concentrations exceed the European Union Directive's recommended limit of 0.05 mg/kg (Table 5). Potential sources of Cd in Lake Kivu include regional mining activities on steep slopes, contamination in fertilizers, wastewater, and solid waste from Cd‐containing products (Authman 2015). In comparison, a study assessing tissue metal distribution in fish species from Saudi Arabia reported Cd concentrations in *Oreochromis niloticus* ranging from 1.6 to 1.88 mg/kg, which is slightly higher than the levels found in this study (Mahboob et al. 2014). Another study in Ethiopia reported by Andemo Kotacho et al. (2024) found no detectable levels of Cd in *Oreochromis niloticus* . Additionally, a study in Malaysia reported by Ishak et al. (2020) found a Cd concentration of 0.004 mg/kg in fish.

### 3.4 Copper (Cu)

The average Cu concentration in fish from Lake Kivu was 0.39 mg/kg, with *Oreochromis niloticus* showing the highest level at 0.76 mg/kg and *Clarias liocephalus* showing the lowest level at 0.28 mg/kg.

Analysis of variance (ANOVA) revealed no significant differences in copper (Cu) concentrations among the fish species studied (*F* = 0.998, *p* = 0.477), indicating that the mean Cu levels were comparable across all species. Consistently, Tukey HSD post hoc tests showed that all pairwise comparisons had confidence intervals including zero and adjusted *p*‐values near 1, confirming that Cu concentrations did not differ significantly between any of the species. These findings suggest that species identity does not have a discernible effect on Cu accumulation in the sampled fish, and the minor variations observed are likely attributable to random variation rather than biological differences.

Cu values are below the maximum permissible limit set by the FAO/WHO, which is 3 mg/kg (Table 5). Despite being within permissible limits, Cu concentrations in Lake Kivu fish were higher compared to those reported in other studies. For instance, a study on Taihu Lake in China reported Cu concentrations ranging from 0.034 to 0.037 mg/kg (Rajeshkumar and Li 2018). Similarly, in Lake Ińsko and Lake Wisola in Northwestern Poland, Cu concentrations in fish were found to be 0.14 and 0.19 mg/kg, respectively (Rajkowska and Protasowicki 2013). However, in Lake Rukwa, Tanzania, Cu concentrations in fish ranged from 1.52 to 3.71 mg/kg, while in the Egyptian Red Sea, Cu concentrations in fish ranged from 9.07 to 9.24 mg/kg, higher than those in Lake Kivu (Abbas and Alnasser 2025; Mapenzi et al. 2020).

Potential sources of Cu pollution in Lake Kivu include wastewater from various activities involving Cu use, and applications of Cu in fertilizers, pesticides, food additives, electroplating, wood preservatives, and azo dyes (Ishchenko 2018).

### 3.5 Chromium (Cr)

The average Cr concentration in fish from Lake Kivu was 3.01 mg/kg, with *Oreochromis niloticus* having the highest concentration at 3.83 and *Lamprichthys tanganicanus* the lowest at 2.58 mg/kg.

The ANOVA results for chromium (Cr) concentrations among the fish species showed no significant differences (*F* = 0.291, *p* = 0.986), indicating that Cr levels were comparable across all species. Similarly, the Tukey HSD post hoc tests revealed that all pairwise comparisons had confidence intervals including zero and adjusted *p*‐values close to 1, confirming the absence of statistically significant differences between any species. These results suggest that species identity does not have a discernible effect on Cr accumulation in the sampled fish, and any minor variations observed are likely due to random variation rather than true biological differences.

Cr levels exceed the maximum permissible limit of 0.15 mg/kg set by the FAO/WHO (Table 5). Compared to other studies, the Cr concentrations in Lake Kivu were lower than those found in a study conducted in Bangladesh on cultivated fishes, where levels ranged from 4.71 to 8.98 mg/kg (Saha et al. 2021). Similarly, higher Cr concentrations were detected in fish from the Lower Meghna River in Bangladesh, with levels reaching 7.34 mg/kg (Hossain et al. 2022). Conversely, other studies reported lower Cr concentrations. For instance, fish from a tropical estuary in Bangladesh had a Cr concentration of 0.91 mg/kg (Mohiuddin et al. 2022), and a study on fish from a reservoir in China found Cr levels at 0.173 mg/kg (Zheng et al. 2007). Sources of Cr in Lake Kivu include volcanic activity releasing Cr into the atmosphere, which is then deposited into water bodies, as well as wastewater and solid waste from human activities (Yang et al. 2019).

### 3.6 Manganese (Mn)

The average Mn concentration in fish from Lake Kivu was 0.33 mg/kg, with the highest concentration found in *Oreochromis niloticus* at 0.68 mg/kg and the lowest in *Clarias liocephalus* at 0.22 mg/kg.

The ANOVA results for manganese (Mn) concentrations across the fish species revealed statistically significant differences (*F* = 3.855, *p* = 0.00193), indicating that Mn accumulation varies among species. The Tukey HSD post hoc comparisons identified several significant pairwise differences, most notably involving *Oreochromis niloticus* and *Varicorhinus platystomus* , which consistently showed higher Mn concentrations compared to multiple other species. These findings suggest that species identity plays a significant role in Mn accumulation in the sampled fish, and the observed variation is likely driven by species‐specific physiological or ecological traits rather than random variation.

Mn levels are below the FAO/WHO maximum permissible limit of 1 mg/kg (Table 5). When compared to other studies, the Mn concentrations in Lake Kivu are within the range reported for fish from the Mediterranean Sea along the Gaza Coast in Palestine (0.26 to 1.26 mg/kg) (Zaqoot et al. 2017). An assessment of trace metals in fish from the Khuzdar Balochistan River in Pakistan reported Mn concentrations ranging from 0.22 to 1.9 mg/kg, similar to those in Lake Kivu (Gurganari et al. 2024). However, the Mn concentrations in fish from Lake Kivu were lower than that found in fish from the Red Sea in Egypt, which ranged from 0.10 to 0.29 mg/kg (El‐Moselhy et al. 2014). Sources of Mn in Lake Kivu can be the weathering of Mn‐containing rocks, mining activities, and wastewater from various sources (O'Neal and Zheng 2015).

### 3.7 Mercury (Hg)

Hg had an average concentration of 0.42 mg/kg, with the lowest concentration at 0.07 mg/kg found in *Haplochromis rubescens* and the highest concentration found in *Oreochromis niloticus* at 2.53 mg/kg.

The ANOVA results indicate a highly significant difference in mercury (Hg) concentrations among the fish species analyzed (*F*(12,26) = 23.62, *p* < 0.001), showing that species identity strongly influences Hg accumulation. The subsequent Tukey HSD test reveals that most pairwise differences are not statistically significant, except for comparisons involving *Oreochromis niloticus* , which consistently show significantly higher Hg levels compared to nearly all other species (*p* < 0.001).

Hg value greatly exceeds the maximum permissible limit of 0.5 mg/kg set by the European Union Directive (Table 5). The Hg concentration in fish from Lake Kivu was higher than the values reported in a study on trace metal accumulation in fish from southern Italy, which ranged from 0.03 to 0.64 mg/kg (Barone et al. 2021). Similarly, Hg levels in Lake Kivu fish were higher than those reported in fish from southwestern Nigeria, which ranged from 0.24 to 0.44 mg/kg (Emmanuel and Samuel 2009). The sources of Hg in Lake Kivu are varied, including erosion from the watershed, volcanic emissions, and anthropogenic sources such as wastewater discharge (Nsabimana et al. 2020). In reducing environments, ionic Hg is readily methylated by various bacteria, particularly sulfate‐reducing bacteria, to form methyl mercury (Al‐Sulaiti et al. 2022). Methyl mercury is then strongly bioaccumulated and biomagnified through the food chain, leading to higher concentrations at higher trophic levels (Driscoll et al. 2013).

Overall, the trace element concentrations in Lake Kivu fish are generally within the ranges observed in similar freshwater ecosystems, though Cd and Hg show relatively higher concentrations compared to certain studies. We found Pb levels below the limit of quantification, which is lower than in other studies such as Saha et al. (2021) and Mahboob et al. (2014), where Pb concentrations ranged from 1.03 to 7.79 and 3.05 to 5.12 mg/kg, respectively.

The Cd levels in Lake Kivu fish (0.661–1.431 mg/kg) are higher than those reported in some studies (e.g., El‐Moselhy et al. 2014; Zaqoot et al. 2017; Rajeshkumar and Li 2018), where values ranged from 0.02 to 0.51 mg/kg. However, concentrations are within the range found in Mahboob et al. (2014) (1.60–2.48 mg/kg) and comparable to Saha et al. (2021) (0.87–1.35 mg/kg). The Mn concentrations in Lake Kivu fish (0.204–1.455 mg/kg) are comparable to those reported in other studies, such as Gurganari et al. (2024) (0.22–1.9 mg/kg) and Zaqoot et al. (2017) (0.26–1.26 mg/kg). However, they are significantly lower than those in Mahboob et al. (2014), where Mn levels reached 19.81–37.18 mg/kg.

The Cr levels in Lake Kivu fish (2.422–6.406 mg/kg) are relatively high compared to some studies, such as Zheng et al. (2007) (0.07–0.28 mg/kg) and Mohiuddin et al. (2022) (0.059–1.605 mg/kg). However, concentrations fall within the range reported by Saha et al. (2021) (4.71–8.98 mg/kg). The Hg concentrations in Lake Kivu fish (0.029–5.413 mg/kg) show significant variability. Values are higher than those reported by Emmanuel and Samuel (2009) (0.24–0.44 mg/kg) and Barone et al. (2021) (0.03–0.64 mg/kg) but comparable to Hossain et al. (2022) (0.72–2.9 mg/kg).

The Cu levels in Lake Kivu fish (0.255–1.592 mg/kg) are within the range observed in studies like El‐Moselhy et al. (2014) (0.22–0.36 mg/kg) and Gurganari et al. (2024) (0.04–1.2 mg/kg). However, concentrations are significantly lower than in Mahboob et al. (2014) (18.71–43.61 mg/kg) and Hossain et al. (2022) (30.29–48.59 mg/kg). We found that arsenic concentrations were below the limit of quantification, whereas some other studies, such as Mahboob et al. (2014), reported higher concentrations (3.25–7.31 mg/kg).

### 3.8 Bio‐Concentration Factor (BCF) of Trace Elements in Fishes From Lake Kivu

The bio‐concentration factors (BCFs) of trace elements in various fish species from Lake Kivu are presented in Table 6. The table indicates the BCFs for Cd, Cu, Cr, Mn, and Hg in different fish species. Note that the BCFs for lead and arsenic were not included, as measured concentrations in fish were below the quantification limits.

**TABLE 6:** Bio‐concentration factors of trace elements in fish species.

Scientific names	Local names	Cd	Cu	Cr	Mn	Hg
*Labeo victorianus*	Indugu	115.48	64.06	16.78	0.523	2867
*Tilapia rendalli*	Indoba	120.24	71.88	18.53	0.598	533
*Barbus neumayeri*	Ingege	128.57	56.25	17.68	0.448	1733
*Clarias liocephalus*	Isembe	105.95	43.75	17.91	0.329	1333
*Oreochromis niloticus*	Tilapia	132.14	118.75	21.64	1.016	16,867
*Limnothrissa miodon*	Isambaza	110.71	51.56	16.10	0.404	1333
*Varicorhinus platystomus*	Gakara	109.52	50.00	16.50	0.404	1267
*Barbus apleurogramma*	Imyungu	138.10	56.25	17.23	0.463	1533
*Oreochromis mweruensis*	Bwiza	101.19	51.56	15.42	0.404	1067
*Lamprichthys tanganicanus*	Rwandarushya	111.90	54.69	14.58	0.433	2400
*Haplochromis graueri*	Mupfumu	82.14	51.56	15.88	0.404	1333
*Haplochromis rubescens*	Gishoga	111.90	59.38	16.78	0.478	467
*Haplochromis scheffersi*	Baraka	116.67	54.69	16.33	0.433	3533

*Note:* The bio‐concentration factors (BCFs) of trace elements in various fish species from Lake Kivu. The BCF values indicate the extent to which these trace elements accumulate in fish tissues relative to their concentration in the surrounding water.

*Oreochromis niloticus* exhibited significantly higher bio‐concentration factors (BCFs) for Hg compared to other species, with a value of 16,867 L/kg. *Haplochromis scheffersi* also displayed a high BCF for Hg at 3533 L/kg, followed by *Labeo victorianus* with a BCF of 2867 L/kg. The lowest BCF for Hg was observed in *Haplochromis rubescens* , at 467 L/kg. For Cd, *Barbus apleurogramma* showed the highest BCF of 138.1 L/kg, while *Haplochromis graueri* indicated the lowest BCF of 82.14 L/kg. Among the fish species listed, *Oreochromis niloticus* demonstrated a significantly higher BCF for Cu at 118.75 L/kg, whereas *Clarias liocephalus* exhibited the lowest BCF for Cu at 43.75 L/kg. The highest BCF for Cr was observed in *Oreochromis niloticus* at 21.64 L/kg, and the lowest in *Lamprichthys tanganicanus* at 14.58 L/kg. The highest BCF for Mn was observed in *Oreochromis niloticus* at 1.016 L/kg, and the lowest in *Clarias liocephalus* at 0.329 L/kg.

Hg consistently showed the highest BCF values across all species, regardless of feeding habits, indicating its widespread presence and efficient bioaccumulation in fish. The differences in bio‐concentration factors among the fish species in Lake Kivu can largely be attributed to their feeding habits and trophic levels (Ahmed et al. 2019). Omnivorous fish species can consume a wide variety of food sources, including detritus, algae, and smaller invertebrates, which may contribute to their higher bioaccumulation (Ouro‐Sama et al. 2021). *Oreochromis niloticus* is an omnivorous species and exhibits very high BCF values across all trace elements, especially Hg. This pattern is due to its ability to occupy various trophic levels and feed on both plant material and small aquatic organisms. *Haplochromis scheffersi* , another omnivorous species, shows moderate to high BCF values for most trace elements studied, reflecting its varied diet and potential exposure pathways in the environment. Planktivores and herbivores generally exhibit lower BCFs, reflecting their position at lower trophic levels (Jiang et al. 2021). *Haplochromis graueri* is primarily herbivorous and indicated relatively lower accumulation across multiple trace elements compared to other species.

### 3.9 Target Hazard Quotient and Hazard Index of Trace Elements in Fishes From Lake Kivu

**FIGURE 2:**
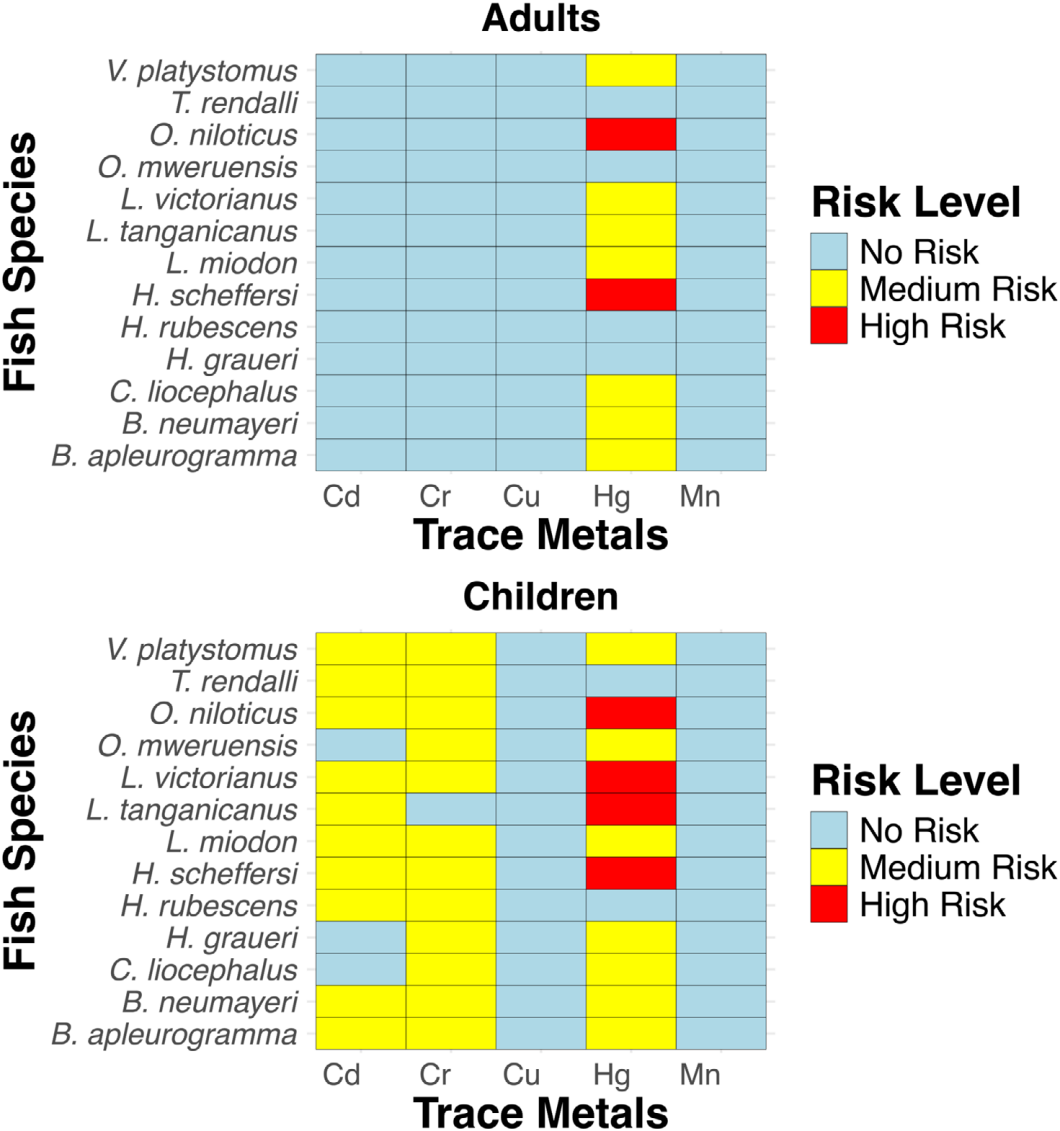
Target hazard quotient (THQ) of trace metals in adults and children for different fish species. The risk levels are color‐coded: Red indicates high risk, and blue represents low risk. Notably, *Oreochromis niloticus* ( *O. niloticus* ) exhibits a high mercury risk for both adults and children, emphasizing potential health concerns related to its consumption. The classification was based on U.S. Environmental Protection Agency, regional screening levels (RSLs), user's guide (USEPA 2021).

The Target Hazard Quotient (THQ) is a measure used to evaluate the potential non‐carcinogenic health risks associated with trace element exposure through fish consumption (Kortei et al. 2020). The U.S. Environmental Protection Agency (EPA) and the World Health Organization (WHO) recommend that THQ values remain below 1 for safe consumption. A THQ value above 1 suggests that long‐term consumption of contaminated fish may pose health risks, while a THQ below 1 is generally considered safe for human consumption over a lifetime (Chen et al. 2011). The Hazard Index (HI) represents the combined effect of multiple trace elements. An HI greater than 1 suggests an increased likelihood of adverse health effects from cumulative exposure. Key trace elements of concern in this study include Cd, Cr, Mn, and Hg, which can cause kidney damage, neurological disorders, and developmental issues with prolonged exposure. The Target Hazard Quotient (THQ) and Hazard Index (HI) for adults and children are presented in Figure 2.

From the THQ values for adults, most fish species show THQ values below 1 for individual trace elements except for Hg, which has a significantly high THQ and high total HI values. The highest THQ Hg value for adults was observed for *Oreochromis niloticus* (14.457), followed by *Haplochromis scheffersi* (3.29) and *Labeo victorianus* (2.457); these values suggest that consumption of these fish species poses significant health risks. The THQ values for Cd in adults ranged from 0.394 to 0.663, suggesting no immediate health risk. The THQ values for Cd in children range from 0.773 to 1.299. The THQ values for Cu in both adults and children were below 1, indicating no significant health risk. The THQ values for Cr in adults range from 0.491 to 0.730, again indicating no significant health risk. The THQ values for Cr in children range from 0.963 to 1.430. The THQ values for Mn in both adults and children were below 1, indicating no significant health risk. *Oreochromis niloticus* exhibits the highest Health Index (HI) for adults (15.83) followed by *Haplochromis scheffersi* (4.15) with the lowest HI value found in adults found in *Haplochromis rubescens* (1.51).

For children, the THQ values for Cd, Cr, and Hg exceeded 1, with Hg exhibiting the highest THQ values. The highest THQ Hg value for children was observed for *Oreochromis niloticus* (28.336), followed by *Haplochromis scheffersi* (5.936) and *Labeo victorianus* (4.816). Long‐term exposure to Hg can impair cognitive development in children. Cd and Cr exposure can increase the risk of kidney damage and potential carcinogenic effects for children. The highest HI values for children were observed for *Oreochromis niloticus* (31.04), followed by *Haplochromis scheffersi* (8.12) and *Labeo victorianus* (7.03). These values suggest that the consumption of these fish species poses significant health risks for children, who are more vulnerable to trace element toxicity.

Children are at a higher risk for trace element toxicity compared to adults, as indicated by their higher THQ values. More than 75% of the analyzed fishes were found to have a risk of Hg higher than 1 for adults, while more than 80% of the analyzed fishes indicate a risk for Hg higher than 1 for children. The significant THQ values for Hg in *Oreochromis niloticus* highlight a severe health risk for both adults and children. This level suggests a need for urgent measures to reduce Hg contamination in these fish to facilitate decreases in exposure.

Mitigating the risks associated with consuming contaminated fish from Lake Kivu requires an integrated approach involving public education, regulatory measures, and environmental interventions. Consumption advisories should be established and enforced based on contamination levels, with recommendations to limit the intake of Tilapia and other species known to accumulate high levels of trace elements.

Special attention should be given to vulnerable populations such as children, pregnant women, and nursing mothers. Community education campaigns are essential to raise awareness about the health risks of consuming contaminated fish and to promote safe preparation practices, such as removing the skin, fat, and internal organs where toxins tend to concentrate. It is equally important to ensure that consumption advisories and preparation guidelines are accessible to the public through signage near Lake Kivu, local government websites, and community outreach programs.

To address the sources of contamination, measures such as establishing buffer zones around the lake to reduce agricultural and industrial runoff and implementing wastewater treatment systems to mitigate pollution from mining activities and other anthropogenic sources should be prioritized. Additionally, environmental remediation efforts, such as introducing non‐contaminated fish species or fish species that are herbivorous or omnivorous (limiting trophic transfer of contaminants), or managing populations of fish that bioaccumulate trace elements, can further help reduce risks. By combining these strategies, it is possible to protect the health of communities relying on Lake Kivu while ensuring the long‐term sustainability of its ecosystem.

### 3.10 Carcinogenic Risk

Carcinogenic risk values are used to estimate the probability of an individual developing cancer over a lifetime due to exposure to a carcinogenic substance (Onoyima et al. 2022). According to the guidelines of the U.S. Environmental Protection Agency (EPA) and the World Health Organization (WHO), a risk value above 1 in 1,000,000 (1 × 10^−6^) is considered significant and may warrant regulatory action. However, values above 10^−4^ may indicate a significant carcinogenic risk (El Fadili et al. 2024).

**FIGURE 3:**
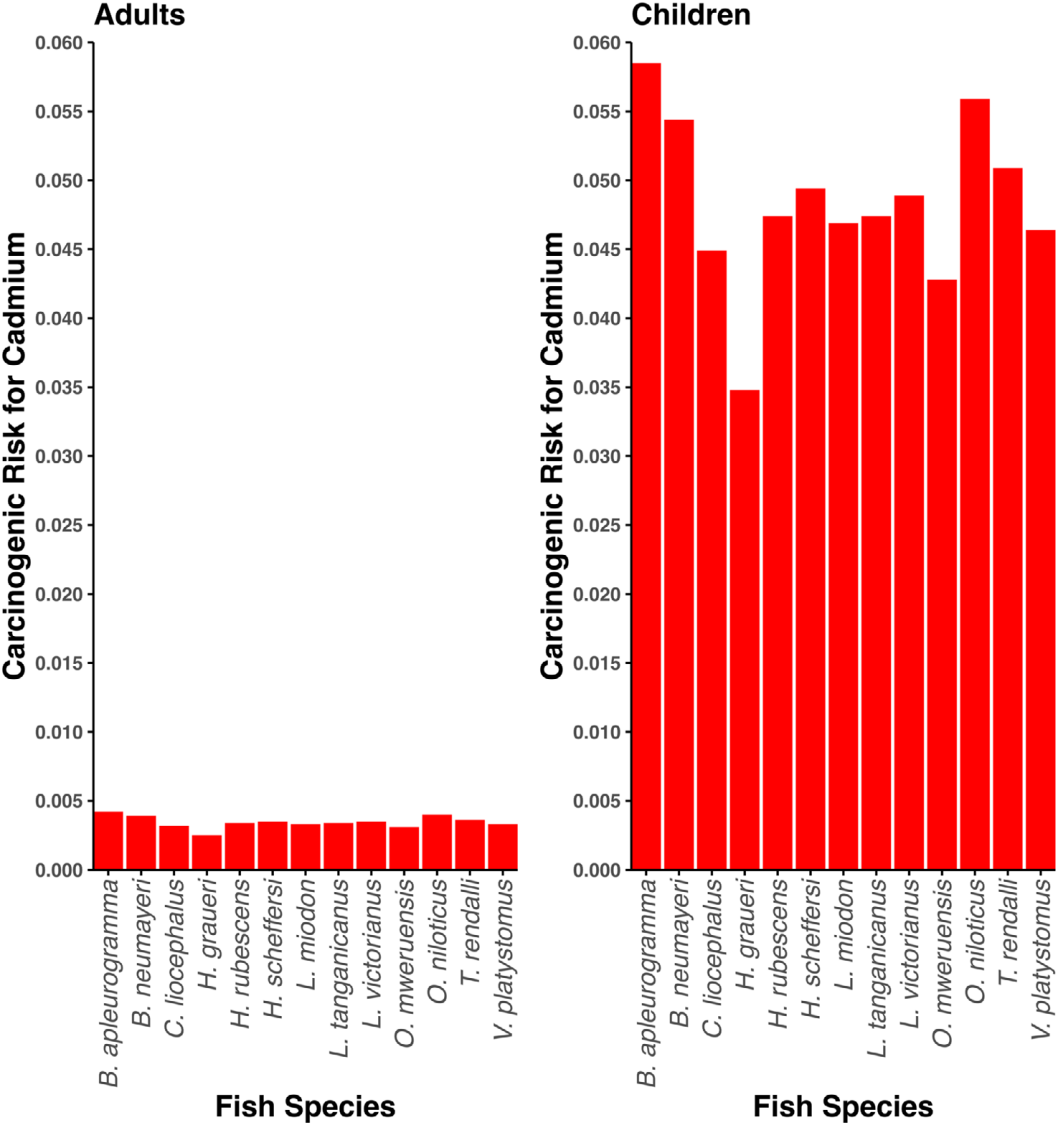
Carcinogenic risk (CR) for cadmium in adults and in children across fish species. This figure illustrates the estimated carcinogenic risk for cadmium exposure in adults and children based on fish consumption from Lake Kivu. The CR values are calculated using cadmium concentrations in fish tissues and dietary intake parameters. Higher risk values indicate a greater potential health concern, particularly for vulnerable populations such as children.

Among the analyzed trace elements, Cd, Cr, and As are classified as human carcinogens by the International Agency for Research on Cancer (IARC) (IARC 2012). However, only the carcinogenic risk of Cd was calculated in the study, as the concentrations of As were below the limits of quantification, and Cr was measured as total Cr rather than Cr (VI) which is the carcinogenic form. Carcinogenic risks are presented in Figure 3.

The carcinogenic risk posed by Cd exposure through the consumption of fish species is considerably higher for both adults and children than internationally accepted safety thresholds. For adults, the carcinogenic risk values ranged from 0.0025 to 0.0042, all of which significantly exceed the commonly accepted safety threshold of 0.00001 for carcinogenic risk. These results indicate that prolonged consumption of these fish species could pose a significant health concern. The highest carcinogenic risk for adults was observed in *Barbus apleurogramma* (0.0042), followed closely by *Oreochromis niloticus* (0.0040), while *Haplochromis graueri* presented the lowest risk at 0.0025. Despite this variation among species, all fish studied were associated with Cd levels far above the acceptable limit.

The situation is even more concerning for children, who are more vulnerable to toxic exposures due to their developing physiology and higher intake of food per unit body weight compared to adults. Carcinogenic risk values for children in this study ranged from 0.0348 for *Haplochromis graueri* to 0.0585 for *Barbus apleurogramma* . These values are more than 1000 times higher than the safety threshold of 0.00001, highlighting a substantial health concern. Notably, the same fish species ( *Barbus apleurogramma* and *Oreochromis niloticus* ) that posed the greatest risks to adults were also associated with the highest risks for children, with carcinogenic risk values of 0.0585 and 0.0559, respectively.

The presence of Cd in these fish species and the associated carcinogenic risks highlight the need for regular monitoring and regulatory measures to control Cd levels in water bodies and aquatic life. The consistent presence of Cd and associated risks across all fish species studied suggests a need for ongoing vigilance and proactive measures to ensure food safety and public health.

Given that approximately 2 million people live around Lake Kivu and 20% of households consume fish, the estimated number of people exposed to carcinogenic risk is around 500,000. This estimate is conservative, as not all individuals will be consuming the most contaminated species.

## 4 Conclusions

In this study, we provide valuable insight into the levels of trace elements contamination in fish from Lake Kivu, Rwanda, with average concentrations at 0.956 mg/kg for Cd, 0.368 mg/kg for Cu, 2.964 mg/kg for Cr, 0.307 mg/kg for Mn, and 0.325 mg/kg for Hg. In this study, Nile Tilapia fish ( *Oreochromis niloticus* ) were found to accumulate greater concentrations of trace elements compared to other fish species in Lake Kivu, with a BCF value of 45,586 for Hg, 10.35 for Cu, 9.27 for Cd, 2.09 for Cr, and 0.09 for Mn. The high bio‐concentration factor (BCF) values for certain fish species, such as *Oreochromis niloticus* (Tilapia), emphasize the need for immediate action to reduce trace element exposure and protect the health of communities relying on Lake Kivu fish as a primary protein source. The results indicate that while some fish species have concentrations of certain trace elements below regulatory limits, others, such as Cd and Hg, exceed these limits, posing potential health risks, particularly for children. The bioaccumulation factor (BCF) and target hazard quotient (THQ) values highlight the varying degrees of trace element accumulation and associated health risks across different fish species. For adults, over 75% of the analyzed fish showed an Hg risk exceeding 1, while for children, this risk was observed in over 80% of the analyzed fish. The risk depends on the quantity consumed, and it is likely to be very low as the general consumption rate of fish in Rwanda is still very low. However, for people living near the lake and the families of fishermen who consume regularly a considerable quantity of fish, the risk can be high. The sample size and species diversity were limited, and seasonal variations in trace element concentrations were not considered. It is recommended to conduct more extensive and regular monitoring of trace element concentrations in Lake Kivu fish, considering seasonal variations and a wider range of species. Future research on long‐term monitoring of trace element levels in both water and fish populations should be conducted to evaluate the sensitivity of different fish species to trace element pollution, the cumulative ecological impacts on biodiversity and ecosystem functions, and the role of specific anthropogenic sources like mining and industrial discharge—including natural sources on—fish contamination. These efforts will help provide a comprehensive understanding of trace element pollution and long‐term risks to aquatic ecosystems and human health.

## Author Contributions

**Valens Habimana:** conceptualization (equal), data curation (equal), formal analysis (equal), investigation (equal), methodology (equal), software (equal), validation (equal), writing – original draft (equal), writing – review and editing (equal). **Svetlana Gaidashova:** methodology (equal), resources (equal), writing – review and editing (equal). **Egide Kalisa:** methodology (equal), resources (equal), writing – review and editing (equal). **Antoine Nsabimana:** conceptualization (equal), funding acquisition (equal), investigation (equal), methodology (equal), project administration (equal), supervision (equal), writing – review and editing (equal). **Christopher A. Scholz:** resources (equal), writing – review and editing (equal). **Charles T. Driscoll:** resources (equal), writing – review and editing (equal).

## Ethics Statement

All study methods were carried out following relevant guidelines and regulations.

## Conflicts of Interest

The authors declare no conflicts of interest.

## Data Availability

The data that support the findings of this study are available from the corresponding author upon reasonable request.
